# Use of dementia care technologies to increase engagement and reduce behavioural and psychological symptoms in people with dementia in a specialist dementia care ward

**DOI:** 10.3389/frdem.2026.1836528

**Published:** 2026-07-14

**Authors:** Catherine Ames, Tim To, Craig Whitehead, Madison Chapman, Julian Soriano, Lisa Bird, Kate Laver

**Affiliations:** 1 Division of Rehabilitation, Aged and Palliative Care, Southern Adelaide Local Health Network, Adelaide, SA, Australia; 2 College of Medicine and Public Health, Flinders University, Adelaide, SA, Australia; 3 College of Nursing and Health Sciences, Flinders University, Adelaide, SA, Australia

**Keywords:** agitation, assistive technologies, behaviour, dementia, tailored activities

## Abstract

**Introduction:**

Behavioural and psychological symptoms of dementia (BPSD) are common and challenging to manage in hospital settings. BPSD can be exacerbated by environmental factors, patient disengagement, and unmet needs. Dementia care technologies offer a promising way to engage individuals in meaningful activities and reduce BPSD. Despite growing interest, evidence remains limited, particularly in clinical settings and among individuals with advanced dementia, highlighting the need for further research into real-world effectiveness and uptake. The aim of this study was to evaluate the effect of three dementia care technologies on patient engagement and behaviour in a specialist dementia care hospital ward.

**Methods:**

A pre-post design involving people with dementia admitted to a specialist dementia care ward was implemented. Participants were invited to use technologies available on the ward (a virtual reality program, the HUG™ device, or the Tovertafel) with the support of an allied health assistant. Engagement and behaviour of participants was rated before, during, and shortly after technology use. Ward-level data on behavioural incidents and medication administration were analysed to explore the wider impact of technology use.

**Results:**

A total of 182 sessions were completed using one of the three technologies. Participants included people who were considered to have severe BPSD and few people declined use. There were significant improvements in all patient behaviours during and immediately after technology use for all three technologies. Analyses suggested that following implementation of the technologies, there was a significant change in trend for serious behavioural incidents.

**Discussion:**

Dementia care technologies appear feasible and effective in reducing BPSD in the short term for a range of people with dementia. Dementia care technologies can be used to offer individualised activity experiences in a specialist dementia care ward.

## 1 Introduction

Behavioural and psychological symptoms of dementia (such as agitation, anxiety, aggression, and repetitive questioning) are very common, affecting an estimated 60% of older people with dementia in hospital settings (Anantapong et al., 2025). The underlying causes of behavioural and psychological symptoms of dementia (BPSD) are complex and multifaceted and include neurodegeneration, environmental contributors (e.g., under or overstimulation), unmet needs (e.g., purposeful activity, social relationships) and biological stressors (e.g., thirst, pain) (Lyketsos, 2007). Behavioural and psychological symptoms of dementia can be distressing for the person experiencing them and difficult to manage in hospital settings where disorientation, change in routine, boredom, lack of purpose, and lack of intricate knowledge of the person can exacerbate symptoms (White et al., 2017). Consequently, people with dementia are often admitted to specialist inpatient dementia care wards that provide dedicated staff with training in dementia care, a supportive and dementia-friendly environment, and routines and strategies in place to manage behaviours (McCausland et al., 2019).

There is increasing recognition that identifying, prescribing, and implementing tailored activities for people with BPSD can be beneficial for people with a range of symptoms, ranging from apathy to aggression (Jeong et al., 2023; Lu et al., 2021; Trahan et al., 2014). However, offering suitable activities for people with dementia in hospital settings can be difficult due to varied abilities within patients, different leisure interests, impaired cognitive and communication skills, infection control issues, and limited staffing available to support activity engagement. Consequently, a range of different dementia care technologies have been developed with the aim of preventing and reducing BPSD. These include devices for reminiscence support (digital cameras, apps with photos and music), social robots, communication aids, games, and virtual reality programs (Goodall et al., 2021). These technologies facilitate engagement in interesting activities that match the person’s cognitive, physical, and social abilities and promote connection to others.

Evidence to date suggests that dementia care technologies can be beneficial in improving relationships between the person with dementia and others, improving behaviour, and positively impacting wellbeing, however, the field is relatively new and few studies currently exist (Goodall et al., 2021; D’Cunha et al., 2019; Hirt et al., 2021; Montgomery et al., 2025). A systematic review examining technologies to engage people with dementia in meaningful activities identified 29 studies with a total of 231 participants across all studies and inclusion of participants ranging from mild to advanced dementia (Goodall et al., 2021). Case studies were the most used study design and most technologies (in *n* = 18 studies) were facilitated by family members and implemented within the homes of community-dwelling participants. Key challenges highlighted in the review were the cost of devices, the challenge of implementing devices in busy environments where staff have limited time to facilitate technology use, and the applicability and feasibility of using devices with people with advanced dementia (Goodall et al., 2021).

Dementia care technologies are widely promoted within the aged care and dementia support sectors, yet for many health and aged care organisations, the potential value of investing in these innovations remains uncertain. Organisations require information about the efficacy, comparable benefits of the various devices available, ease of use, and uptake in dementia care settings. While individual dementia care technologies have been evaluated in a range of small studies (Goodall et al., 2021; Montgomery et al., 2025; Konrad et al., 2024; Appel et al., 2024), few studies have taken place in clinical settings or with people with advanced dementia and therefore ‘real-world’ data on effectiveness and uptake is lacking. A recent Australian study showed that use of a virtual reality program was feasible with people with dementia in a hospital setting however, most of their participants (93%) were classified at Levels 1–4 in regards to the severity of their symptoms of BPSD (Montgomery et al., 2025), reflecting that they had mild to moderate severity symptoms.

The aim of this study was to evaluate the effect of three dementia care technologies on patient engagement and behaviour in a subacute specialist dementia care ward.

## 2 Methods

### 2.1 Study design

This study used a pre-post design. People with dementia who participated in the study used one of the three technologies available on the ward [a virtual reality program from NeuronsVR (NeuronsVR, 2025), HUG^™^ (LAUGH HB, 2025) or the Tovertafel (Tover, 2025)] with the support of an allied health assistant. Observational data of engagement and behaviour of participants were collected before, during, and after technology use to determine the effect of the technologies on individual participants. Ward-level data were collected to explore the wider impact of technology use.

### 2.2 Setting

This study was conducted in the Specialist Advanced Dementia Unit (SADU) within Southern Adelaide Local Health Network (SALHN). The 12-bed unit admits people with dementia and changed behaviours and provides multidisciplinary care. Most of the people admitted to the ward are discharged to a residential care home when a suitable placement becomes available. Participants were recruited between June 2024 and November 2024, at which time the median length of stay was 80 days. There were fourteen admissions to the unit during the study period. The study received ethics approval from the Southern Adelaide Clinical Human Research Ethics Committee (ID: 01.24).

### 2.3 Participants and consent

Patients admitted to the ward were invited to participate in this study, with inclusion criteria being diagnosis of dementia, nursing or allied health staff identifying the patient as appropriate for intervention, and the patient providing verbal assent. There were no specific exclusion criteria. Participants provided verbal assent to participate as written consent was deemed to be too burdensome for this population. Verbal assent is frequently used in dementia research to allow opportunities for people with dementia to participate in research (Black et al., 2010). The allied health assistant who sought assent used verbal, facial, and body language cues to determine assent or dissent, as recommended in the literature (Black et al., 2010). Posters were placed around the unit to ensure family members understood the nature of the research being undertaken. Identifiable personal information, such as name or medical records identifier number, was not collected from participants and therefore data were recorded in a spreadsheet per consultation rather than by participant.

### 2.4 Intervention

Eligible patients were approached by an allied health assistant directly employed three days per week (Monday, Tuesday, Wednesday) during business hours as part of this project and invited to participate in using one of the available technologies. There were no specific patient exclusion criteria. All staff have access to the technologies as part of usual care however they are rarely used, an allied health assistant was specifically employed to use and demonstrate the technologies’ potential benefit on the unit to improve uptake and collect data. Participants who provided assent were supported by the allied health assistant who used modelling, cues, and encouragement to facilitate technology use. Technologies were selected for individuals with consideration of their symptoms; for example, patients who appeared anxious were offered HUG and patients who appeared bored or appeared to be seeking social interaction were offered virtual reality or the Tovertafel. The allied health assistant supervised the patient during use to ensure safety and to monitor for adverse effects (such as motion sickness). The three technologies available on the ward are outlined below.

#### 2.4.1 Virtual reality

The NeuronsVR (2025) headset and program were used to offer participation in a virtual reality experience. These headsets are specifically designed for aged care settings and for people with dementia and include a custom-built head mounted device (which is easy to apply, designed for comfort, and can be worn over glasses). The unit purchased two devices as well as a 12-month subscription to NeuronsVR which provides access to a diverse range of therapy experiences, for example, watching an opera or feeding seagulls at the beach. The allied health assistant supported participants to choose a therapy experience of interest and put on the headset. The allied health assistant remained with the participants during the therapy experience, prompting them to view different elements of the scene and discuss what they were viewing.

#### 2.4.2 Hug

The HUG^™^ is a soft doll (LAUGH HB, 2025) with sensory features including long weighted limbs, a beating heart, and a music player. HUG^™^ is designed to provide comfort to individuals who feel anxious, lonely, or distressed (LAUGH HB, 2025). The allied health assistant offered the HUG to patients, assisting participants to wrap the doll’s arms around them and play calming music.

#### 2.4.3 Tovertafel

The Tovertafel is an interactive game projection system. The system projects interactive light games onto a surface (such as a table) from above. It is specifically designed for people with dementia with games including “clearing autumn leaves” and “feeding birds”. The allied health assistant invited patients to participate in a Tovertafel activity, supporting patients to interact and complete the selected game.

### 2.5 Outcome measures

#### 2.5.1 Patient behaviour

Anonymised demographic data were collected for each participant: gender, age, cognitive score (if available), and Brodaty tier level (Brodaty et al., 2003) for behavioural and psychological symptoms of dementia. The cognitive score and Brodaty tier level were assessed by ward staff as part of their usual care processes. The person’s tier level is assessed upon admission to the unit and reviewed weekly with updates as necessary. The allied health assistant completed an abbreviated version of the INTERACT (Short) questionnaire (Baker et al., 2003; Baker and Dowling, 1995) for each participant in the five minutes before use, during use, and in the five minutes after technology use. The INTERACT questionnaire is a commonly used proxy measure of behavioural and psychological symptoms of dementia where the proxy rates behaviours based on their observations (for example, enjoying self, active, or alert). Each behaviour is rated on a five-point Likert scale in frequency from “not at all” (score 1) to “nearly all the time” (score 5) in the last five minutes (Baker et al., 2003; Baker and Dowling, 1995). Some of the items on the INTERACT questionnaire are behaviours which are considered positive behaviours (such as being happy/content and enjoying self/active/alert) and therefore higher scores are better. Some behaviours on the questionnaire are considered negative behaviours (such as fearful/anxious and bored/inactive) and therefore higher scores are worse.

#### 2.5.2 Ward outcomes

Routinely collected data for number of occasions of “pro re nata” (PRN) medication use (antipsychotics, anxiolytics, and anticonvulsants), number of Code Blacks called (personal threat or security emergency), and number of serious behavioural incidents [measured using reports lodged on the hospital’s Safety Learning System (SLS)] in the unit prior to and during the intervention phase were collected. These data were collected as counts or numbers of occasions per month over the 12-month project period (six months usual care, six months intervention).

### 2.6 Data analysis

Data were uploaded into IBM SPSS (Version 29) for analysis. Descriptive statistics were used to present data on participant characteristics and technology use. We used paired samples t-test (repeated measures) to determine differences before-during and before-after technology use for each item on the INTERACT scale. We conducted an interrupted time series to evaluate the impact of the intervention, introduced in Month 7, on PRN medication use, Code Blacks, and serious behavioural incidents (SLS) over a 12-month period using a segmented linear regression model. The 12-month period included 6 months of usual care followed by 6 months of intervention using the technologies.

## 3 Results

Over the course of the project, participants were invited on 195 occasions to participate in a consultation using one of the technologies. On 182 (93%) occasions, the person consented to use the technology and data for the consultation were obtained. No adverse events during use were reported. Characteristics of participants and technology use are presented in Table 1. Most consultations were with participants who were male with moderate dementia. The most frequently used technology was the virtual reality program.

**Table 1:** Characteristics of consultations.

Characteristics	
Sex of person attending the consultation	25% female, 75% male
Age of person attending (standard deviation)	76.4 (6.2), range 63–90
Cognitive assessment score	MMSE: mean 12.3 (SD 6.7)
Brodaty tier at time of consultation (%)	
Tier 3	15%
Tier 4	48%
Tier 5	19%
Tier 6	0%
Tier 7	19%
Technology used [occasions of service, *n* (%)]	
HUG	36 (20%)
Tovertafel	45 (25%)
Virtual reality	101 (56%)
Average duration of use, mins	
All devices	24.2 (range 1–120)
HUG	50.6 (range 15–120)
Tovertafel	40.3 (range 10–95)
Virtual reality	7.7 (range 2–60)

### 3.1 Patient behaviour

Participant responses to the different technologies used, measured using the abbreviated INTERACT (Short) questionnaire, are presented in Table 2. Participants were often assessed as being anxious and bored in the five minutes prior to technology use. During technology use the severity of these negative behaviours was reduced and the presence of positive behaviours increased. After technology use (in the following five minutes) negative behaviours remained reduced and positive behaviours remained increased, though to a lesser extent than when using the technology. Change scores from before-during and before-after are presented in Table 3. Changes in all behaviours and for all technologies were shown to be statistically significant.

**Table 2:** Participant responses to technology on the INTERACT scale, mean (SD).

Technology use	Happy/content	Fearful/anxious	Attentive/focused	Enjoying self, active, alert	Bored, inactive, sleeping inappropriately
HUG					
Before	1.0 (0.2)	4.9 (0.6)	1.3 (0.8)	3.7 (1.7)	4.9 (0.3)
During	4.9 (0.7)	1.1 (0.2)	4.5 (1.0)	4.9 (0.3)	1.0 (0.2)
After	4.9 (0.3)	1.1 (0.5)	4.6 (1.0)	4.9 (0.3)	1.0 (0.0)
Tovertafel					
Before	1.2 (0.5)	4.7 (0.9)	1.7 (1.1)	4.3 (1.4)	4.8 (0.7)
During	4.8 (0.4)	1.2 (0.5)	4.9 (0.3)	5.0 (0.2)	1.0 (0.0)
After	4.6 (0.9)	1.5 (1.1)	4.6 (0.9)	4.9 (0.3)	1.2 (0.7)
VR					
Before	1.2 (0.5)	4.7 (0.7)	1.2 (0.6)	3.6 (1.8)	4.9 (0.5)
During	4.9 (0.5)	1.1 (0.3)	4.8 (0.8)	5.0 (0.2)	1.0 (0.1)
After	4.9 (0.3)	1.2 (0.6)	4.8 (0.5)	4.9 (0.3)	1.1 (0.3)

**Table 3:** Changes in behaviour during and after technology use according to the INTERACT scale.

Technology use	Happy/content	Fearful/anxious	Attentive/focused	Enjoying self, active, alert	Bored, inactive, sleeping inappropriately
HUG					
Before-during	−3.8** (0.7)	3.8** (0.6)	−3.2** (1.5)	−1.2** (1.8)	3.9** (0.5)
Before-after	−3.9** (0.4)	3.7** (0.7)	−3.2** (1.5)	−1.2** (1.7)	3.9** (0.3)
Tovertafel					
Before-during	−3.6** (0.6)	3.5** (1.0)	−3.3** (1.2)	−0.7* (1.3)	3.8** (0.7)
Before-after	−3.4** (1.2)	3.2** (1.3)	−3.0** (1.5)	−0.7* (1.4)	3.6** (1.0)
VR					
Before-during	−3.7** (0.7)	3.7** (0.8)	−3.6** (1.1)	−1.3** (1.8)	3.9** (0.5)
Before-after	−3.8** (0.6)	3.6** (1.0)	−3.6** (0.9)	−1.3** (1.8)	3.8** (0.6)

*Denotes significance level of <0.05. **Denotes significance level of <0.001.

### 3.2 Ward outcomes

**Figure 1:**
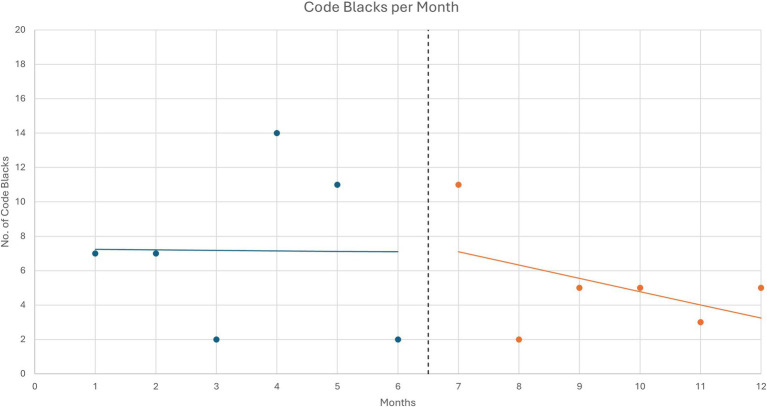
Code blacks per month over time with allied health assistant facilitated technology use introduced month 7.

**Figure 2:**
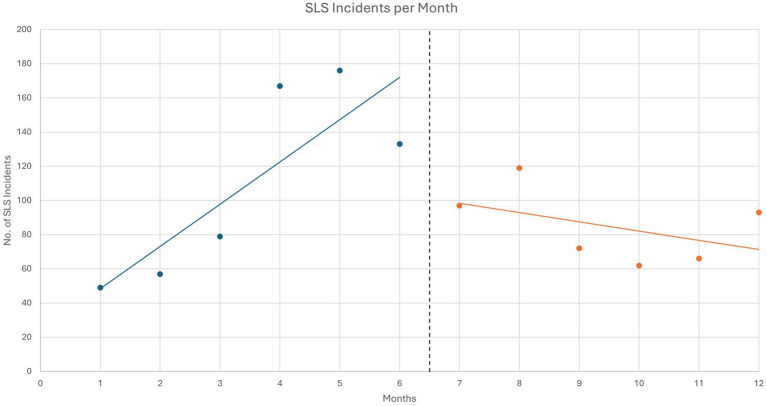
SLS incidents per month over time with allied health assistant facilitated technology use introduced month 7.

The number of Code Blacks and serious behavioural incidents (SLS report) per month are presented in Figures 1, 2. The ward was always at full occupancy during data collection (total of 12 beds). As can be seen, Code Blacks remained relatively consistent over the 12 month period. On average, there were an average of 7.2 Code Blacks per month in the first six months of the data collection period (usual care) and 5.2 Code Blacks per month during the six-month technology intervention phase. Despite this difference, regression analyses showed no significant pre-intervention trend (*p* = 0.98), immediate effect (*p* = 0.89), or change in slope (*p* = 0.63).

There were greater fluctuations in reporting serious behavioural incidents (SLS incidents). On average, there were 110.2 per month reports during the initial six months of data collection followed by 84.8 per month during the six-month technology intervention phase. Regression analyses showed that prior to technology implementation there was a significant (*p* < 0.01) upward trajectory in serious behavioural incidents. While there was no immediate effect at the commencement of the intervention period (*p* = 0.08), there was a significant change in slope during the intervention period (*p* < 0.05), with incidents following a downward trajectory suggesting potential benefit of technology use.

**Figure 3:**
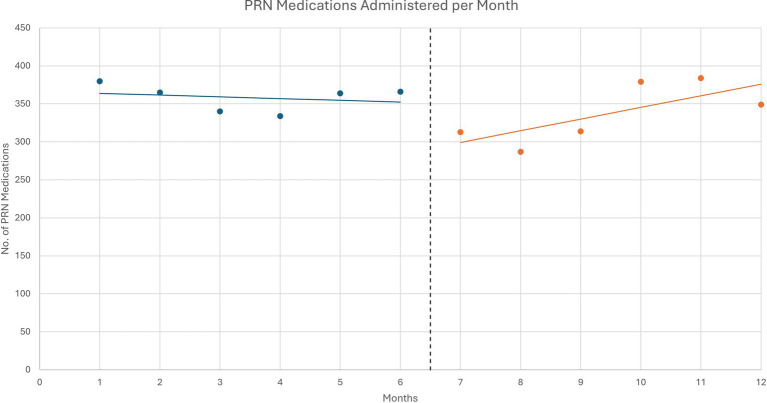
PRN medication use per month over time with allied health assistant facilitated technology use introduced month 7.

Interrupted time series analyses evaluated the impact of the intervention, introduced in Month 7, on PRN use over a 12-month period using a segmented linear regression model (see Figure 3). Prior to implementation the trend in PRN use was not statistically significant (*p* = 0.72), indicating a stable baseline period. There was no immediate effect on PRN use at intervention (*p* = 0.05) nor was there a significant change in trend over the intervention period (*p* = 0.07), suggesting PRN use on the ward was not impacted by the intervention.

## 4 Discussion

This study evaluated the uptake and effects of using three different dementia care technologies on a specialist dementia care ward within a hospital. All three technologies were used and few people with dementia admitted to the ward declined participation when invited. Positive changes in the person’s behaviour were seen during use of all three technologies and benefits appeared sustained for at least five minutes after use. These findings support the implementation of dementia care technologies for people with moderate to severe dementia in specialist dementia care settings.

Of the three devices, the virtual reality program was used the most during this period of data collection. This may be because the device offered the widest range of experiences (e.g., from the opera to the farm) and therefore was most able to be individualised to the person’s interests, an important aspect of dementia care (Brooker, 2003). It may also be that the immersive nature of the virtual reality experience was considered to be more effective in alleviating BPSD. For example, patients using the HUG device were still able to see and hear the activities occurring within their environment whereas people using virtual reality could only see and hear the virtual environment. This sense of presence is an important feature of virtual reality and has also been used to help people with other conditions such as children with anxiety (Arane et al., 2017) and individuals requiring pain management (Viderman et al., 2023). However, while the virtual reality program was used the most, the amount of time that participants spent using the program was the lowest (average 7.7 min) reflecting that most of the VR experiences offered within this program are short and that most people only undertook one experience. Another Australian study which similarly used the NeuronsVR program with participants with lower severity BPSD also reported that the average duration of use was six to seven minutes (Montgomery et al., 2025). The HUG device was used the least of the technologies. A previous study evaluating the HUG in the UK found that the HUG was rejected by some participants, although those that accept the HUG experienced a range of benefits including reduced distress and improved ability for care staff to assist with care (Treadaway et al., 2023).

Uptake of the devices was strong with a part-time allied health assistant using the devices on a total of 182 occasions during a six-month data collection period. There were very few times when patients declined to participate and patients who used the devices included patients who were classified as having Tier 7 severity dementia, meaning they have extreme BPSD that may include physical violence. However, it is important to note that the technologies were used far less prior to the employment of the dedicated allied health assistant. Barriers to routine use amongst other ward staff (such as nursing and allied health) may include lack of time, other care priorities, or lack of confidence in operating the devices. We have attempted to sustain use on the unit through providing clear instructions for use, sharing the benefits experienced by clients, conducting modelling and demonstration sessions, and placing “reminder” posters around the ward however, such high levels of technology use have not been sustained.

Data suggested that the number of serious behavioural incidents (reported using the Safety Learning System) on the ward declined following the introduction of allied health supported technology use. These declines were not seen in Code Blacks or use of PRN medications. There were relatively few Code Blacks on the ward at this time and therefore our data should be interpreted with caution.

This study used before-after observations within individuals so that patients were acting as their own controls. Furthermore, observations were taken before and after by the allied health assistant who conducted treatment sessions and was therefore not blinded to participant intervention. These methods were used for pragmatic reasons in a study that received limited funding and involved participants who are complex to involve in research studies. Furthermore, the presence, company and support of the allied health assistant during technology use may have contributed to the positive findings. People with dementia in institutional settings often report loneliness and boredom (Moyle et al., 2011; Gebhard and Frank, 2024) and spending time with the allied health assistant in itself may have contributed to better mood and reduced boredom even without the use of the technologies. This means that the study is at risk of bias and findings should be interpreted with caution. Another limitation is the relatively small number of consultations despite being one of the largest studies in this field to date and providing useful data on three different technologies.

In conclusion, this study suggests that dementia care technologies can be feasibly applied with good uptake in a specialist inpatient dementia care setting and successfully used with a wide range of people with dementia. We recommend that dedicated staff are required for sustained implementation (for example, an activity coordinator, therapy aid, or an allied health assistant). Dementia care technologies may be considered complementary tools to supporting a person-centred model of care and providing individualised, tailored activities for people with behavioural and psychological symptoms of dementia.

## Data Availability

The datasets presented in this article are not readily available because of the conditions approved by the local human research ethics committee. Requests to access the datasets should be directed to Kate Laver, kate.laver@flinders.edu.au.

## References

[ref1] Anantapong K. Jiraphan A. Aunjitsakul W. Sathaporn K. Werachattawan N. Teetharatkul T. . (2025). Behavioural and psychological symptoms of people with dementia in acute hospital settings: a systematic review and meta-analysis. Age Ageing 54:afaf013. doi: 10.1093/ageing/afaf013, 39888603 PMC11784590

[ref2] Appel L. Appel E. Kisonas E. Lewis-Fung S. Pardini S. Rosenberg J. . (2024). Evaluating the impact of virtual reality on the behavioral and psychological symptoms of dementia and quality of life of inpatients with dementia in acute care: randomized controlled trial (VRCT). J. Med. Internet Res. 26:e51758. doi: 10.2196/51758, 38289666 PMC10865216

[ref3] Arane K. Behboudi A. Goldman R. D. (2017). Virtual reality for pain and anxiety management in children. Can. Fam. Physician 63, 932–934, 29237632 PMC5729140

[ref4] Baker R. Dowling Z. (1995). A new Measure of Response to multi-Sensory Environments. Bournemouth: Research Publication. Research and Development Support Unit, Institute of Health and Community Studies, Bournemouth University.

[ref5] Baker R. Holloway J. Holtkamp C. C. M. Larsson A. Hartman L. C. Pearce R. . (2003). Effects of multi-sensory stimulation for people with dementia. J. Adv. Nurs. 43, 465–477. doi: 10.1046/j.1365-2648.2003.02744.x, 12919265

[ref6] Black B. S. Rabins P. V. Sugarman J. Karlawish J. H. (2010). Seeking assent and respecting dissent in dementia research. Am. J. Geriatr. Psychiatry 18, 77–85. doi: 10.1097/JGP.0b013e3181bd1de2, 20094021 PMC2811536

[ref7] Brodaty H. Draper B. M. Low L. F. (2003). Behavioural and psychological symptoms of dementia: a seven-tiered model of service delivery. Med. J. Aust. 178, 231–234. doi: 10.5694/j.1326-5377.2003.tb05169.x, 12603188

[ref8] Brooker D. (2003). What is person-centred care in dementia? Rev. Clin. Gerontol. 13, 215–222. doi: 10.1017/s095925980400108x

[ref9] D’Cunha N. M. Nguyen D. Naumovski N. McKune A. J. Kellett J. Georgousopoulou E. N. . (2019). A mini-review of virtual reality-based interventions to promote well-being for people living with dementia and mild cognitive impairment. Gerontology 65, 430–440. doi: 10.1159/000500040, 31108489

[ref10] Gebhard D. Frank J. I. (2024). Everyday life and boredom of people living with dementia in residential long-term care: a merged methods study. BMC Geriatr. 24:1049. doi: 10.1186/s12877-024-05641-7, 39736528 PMC11686914

[ref11] Goodall G. Taraldsen K. Serrano J. A. (2021). The use of technology in creating individualized, meaningful activities for people living with dementia: a systematic review. Dementia 20, 1442–1469. doi: 10.1177/1471301220928168, 32475166 PMC8132010

[ref12] Hirt J. Ballhausen N. Hering A. Kliegel M. Beer T. Meyer G. (2021). Social robot interventions for people with dementia: a systematic review on effects and quality of reporting. J. Alzheimer's Dis 79, 773–792. doi: 10.3233/JAD-200347, 33361589 PMC7902949

[ref13] Jeong J. Yoo E.-Y. Pryor L. Kang B.-H. Ha Y.-N. (2023). The effects of a tailored activity program for dementia: a systematic review and meta-analysis. Phys. Occup. Ther. Geriatr. 41, 280–291. doi: 10.1080/02703181.2022.2128972

[ref14] Konrad R. Güttler C. Öhl N. Heidl C. Scholz S. Bauer C. (2024). Effects of the Tovertafel® on apathy, social interaction and social activity of people with dementia in long-term inpatient care: results of a non-controlled within-subject-design study. Front. Neurol. 15:1455185. doi: 10.3389/fneur.2024.1455185, 39677866 PMC11638534

[ref15] LAUGH HB. HUG. (2025). Available online at: https://hug.world/

[ref16] Lu S. Zhang A. Y. Liu T. Choy J. C. Ma M. S. Wong G. . (2021). Degree of personalisation in tailored activities and its effect on behavioural and psychological symptoms and quality of life among people with dementia: a systematic review and meta-analysis. BMJ Open 11:e048917. doi: 10.1136/bmjopen-2021-048917, 34845067 PMC8634002

[ref17] Lyketsos C. G. (2007). Neuropsychiatric symptoms (behavioral and psychological symptoms of dementia) and the development of dementia treatments. Int. Psychogeriatr. 19, 409–420. doi: 10.1017/S104161020700484X, 17346363

[ref18] McCausland B. Patel H. Amin J. Baldwin D. Loughran K. Osman-Hicks V. (2019). A systematic review of specialist inpatient dementia care services versus standard inpatient dementia care in acute hospitals. Aging Clin. Exp. Res. 31, 595–610. doi: 10.1007/s40520-018-1021-y, 30259497 PMC6491402

[ref19] Montgomery A. Teus J. K. Paulik O. Smerdely P. Barton C. Lopez M. R. . (2025). NeuronsVR: virtual reality therapy for people living with dementia during an acute hospital admission–a feasibility study. Geriatr. Nurs. 66:103708. doi: 10.1016/j.gerinurse.2025.103708, 41223733

[ref20] Moyle W. Kellett U. Ballantyne A. Gracia N. (2011). Dementia and loneliness: an Australian perspective. J. Clin. Nurs. 20, 1445–1453. doi: 10.1111/j.1365-2702.2010.03549.x, 21366740

[ref21] NeuronsVR NeuronsVR Australia. (2025). Available online at: https://neuronsvr.com/

[ref22] Tover. Tovartafel. (2025). Available online at: https://www.tover.care/au/

[ref23] Trahan M. A. Kuo J. Carlson M. C. Gitlin L. N. (2014). A systematic review of strategies to foster activity engagement in persons with dementia. Health Educ. Behav. 41, 70S–83S. doi: 10.1177/1090198114531782, 25274714 PMC4454346

[ref24] Treadaway C. Seckam A. Fennell J. Taylor A. (2023). HUG: a compassionate approach to designing for wellbeing in dementia care. Int. J. Environ. Res. Public Health 20:4410. doi: 10.3390/ijerph20054410, 36901428 PMC10001493

[ref25] Viderman D. Tapinova K. Dossov M. Seitenov S. Abdildin Y. G. (2023). Virtual reality for pain management: an umbrella review. Front. Med. 10:1203670. doi: 10.3389/fmed.2023.1203670, 37521355 PMC10382225

[ref26] White N. Leurent B. Lord K. Scott S. Jones L. Sampson E. L. (2017). The management of behavioural and psychological symptoms of dementia in the acute general medical hospital: a longitudinal cohort study. Int. J. Geriatr. Psychiatry 32, 297–305. doi: 10.1002/gps.4463, 27019375 PMC5324689

